# SMITH: a LIMS for handling next-generation sequencing workflows

**DOI:** 10.1186/1471-2105-15-S14-S3

**Published:** 2014-11-27

**Authors:** Francesco Venco, Yuriy Vaskin, Arnaud Ceol, Heiko Muller

**Affiliations:** 1 Department of Electronics, Information and Bioengineering, Politecnico of Milan, Milan 20133, Italy; 2 Center for Genomic Science of IIT@SEMM, Istituto Italiano di Tecnologia (IIT), Milan 20139, Italy

## Abstract

**Background:**

Life-science laboratories make increasing use of Next Generation Sequencing (NGS) for studying bio-macromolecules and their interactions. Array-based methods for measuring gene expression or protein-DNA interactions are being replaced by RNA-Seq and ChIP-Seq. Sequencing is generally performed by specialized facilities that have to keep track of sequencing requests, trace samples, ensure quality and make data available according to predefined privileges.

An integrated tool helps to troubleshoot problems, to maintain a high quality standard, to reduce time and costs. Commercial and non-commercial tools called LIMS (Laboratory Information Management Systems) are available for this purpose. However, they often come at prohibitive cost and/or lack the flexibility and scalability needed to adjust seamlessly to the frequently changing protocols employed.

In order to manage the flow of sequencing data produced at the Genomic Unit of the Italian Institute of Technology (IIT), we developed SMITH (Sequencing Machine Information Tracking and Handling).

**Methods:**

SMITH is a web application with a MySQL server at the backend. Wet-lab scientists of the Centre for Genomic Science and database experts from the Politecnico of Milan in the context of a Genomic Data Model Project developed SMITH. The data base schema stores all the information of an NGS experiment, including the descriptions of all protocols and algorithms used in the process. Notably, an attribute-value table allows associating an unconstrained textual description to each sample and all the data produced afterwards. This method permits the creation of metadata that can be used to search the database for specific files as well as for statistical analyses.

**Results:**

SMITH runs automatically and limits direct human interaction mainly to administrative tasks. SMITH data-delivery procedures were standardized making it easier for biologists and analysts to navigate the data. Automation also helps saving time. The workflows are available through an API provided by the workflow management system. The parameters and input data are passed to the workflow engine that performs de-multiplexing, quality control, alignments, etc.

**Conclusions:**

SMITH standardizes, automates, and speeds up sequencing workflows. Annotation of data with key-value pairs facilitates meta-analysis.

## Background

Next-generation sequencing has become a widespread approach for studying the state and the interactions of bio-macromolecules in response to changing conditions living systems are exposed to. A variety of applications have been developed in the last years that permit studying the locations and the post-translational modifications of proteins bound to DNA (ChIP-Seq), the presence of mutations in DNA (DNA-Seq), the expression level of mRNAs (RNA-Seq), the methylation state of DNA (RRBS), the accessibility of chromatin to transcription factors (DNase-Seq), or the interactions of small RNAs and proteins (CLIP-Seq), to name a few. The protocols for these experiments are very different from each other. However, they all share the high-throughput sequencing step that is necessary to read out the signals. Thus, next-generation sequencing has become a corner stone of modern life-science laboratories and is generally carried out by specialized facilities.

A sequencing facility is confronted with multiple problems. It must handle sequencing requests, process the samples according to the application specified, combine multiplexed samples to be run on the same lane such that de-multiplexing is not compromised and track the state of the sample while it is passing through the sequencing pipeline. They must ensure quality, keep track of reagent barcodes used for each sample, deliver the results to the proper user following de-multiplexing, archive the results and support the users when troubleshooting becomes necessary. Furthermore, the user input at request stage can be erroneous and the facility needs to pinpoint inconsistencies as soon as possible. Considering the central importance of sequencing data, a sequencing facility has to meet these demands under constant pressure to produce results as quickly as possible.

From the user's point of view, the time that passes between placing a sequencing request and obtaining interpretable data can be several weeks. To complicate matters further, the user placing a request is generally a biologist without extensive bioinformatics skills. The biologist collaborates with bioinformaticians who process their data to produce browser embedded tracks that the biologist can view and carry out statistical analyses that help interpreting the data, which adds to the lag time. Some of the analysis steps to be carried out by bioinformaticians can be standardized, however. For example, alignment of the data to a reference genome and the generation of corresponding browser tracks is a routine task. Automation of this step alone frees time for the bioinformaticians who can concentrate on more sophisticated types of analyses, shortens the lag time for the biologist, keeps data organized, and optimizes disk space usage.

All these requirements make a dedicated information management system indispensable. Both commercial and non-commercial software solutions are available. Commercial solutions come at considerable costs, lack transparency, and will only be briefly discussed here. Illumina BaseSpace [1] is a web-based system with easy-to-use interfaces of sample submission, tracking and results monitoring. A plugin-based subsystem of NGS processing tools allows selecting default data analysis pipelines or adding custom ones. Yet, the data must be stored on the BaseSpace cloud. Genologics Clarity LIMS [2] is a highly customisable LIMS with a rich set of options and short deployment time.

There is no shortage of non-commercial, open-source solutions. GnomEx [3] provides extensive solutions for sample submission, sample tracking, billing, access control, data organization, analysis workflows, and reporting for both NGS and microarray data. openBis [4] is a flexible framework that has been adapted to be used for proteomics, high content screening, and NGS projects. The WASP [5] system has been developed to meet the demands of NGS experiments and clinical tests. It provides embedded pipelines for the analysis of ChIP-Seq, RNA-Seq, miRNA-Seq and Exome-Seq experiments. NG6 [6] is based on a compact data model composed of projects, runs, and analyses. The analyses are running in the Ergatis [7] workflow management system and can accept different file types produced by Roche 454 and Illumina HiSeq platforms. SLIMS [8] is a sample management tool for genotyping laboratories. SeqBench [9] has been designed to support management and analysis of exome-sequencing data. The PIMS sequencing extension [10] is based on the Protein Information Management System [11] and has been adapted to be used with Applied Biosystems 96-well plate sequencers and two different types of liquid handling robots. There is also an extension with LIMS functionality to the popular Galaxy workflow engine [12] called Galaxy LIMS [13]. The system supports request submission, offers assistance during flow cell layout, and automatically launches Illumina's CASAVA software [14] to perform de-multiplexing and delivery of the user-specific FASTQ files. Being integrated with Galaxy, the data are automatically available to be processed by analysis pipelines stored in Galaxy. MISO [15] provides a wide set of tools for NGS sample submission, tracking, analysis and visualization and it is distributed as a pre-installed software on a virtual image.

Notwithstanding the availability of different open-source LIMS systems for sequencing applications, finding a solution that meets all the demands of a specific laboratory remains a difficult task. In general, a LIMS should provide support for request submission, store metadata associated to each sample and make them searchable, allow the role-based access control, track samples at all stages of the sequencing pipeline, keep track of reagents used, facilitate administrative tasks such as invoicing, do quality control, integrate seamlessly with the sequencing technology in use, process raw data and deliver them to the requester, apply different workflows to the data as requested by the user, and report the results in a structured fashion. At all stages, the user wants to have feedback regarding the state of their samples. An open-source LIMS must be adapted to the specific infrastructure and the project needs of a research institution, which are in constant evolution. Therefore, the LIMS system must be modified accordingly. The effort needed to adapt an existing LIMS and gain sufficient insight into its code base in order to be able to modify it in a productive fashion must be weighed against the effort of developing an in-house solution. The plethora of available LIMS systems testifies that the latter option is often favoured. From this perspective, it seems that a simple LIMS has more chances of being shared than a more complex one. In any case, there is no LIMS that serves all needs in a simple turn-key solution. Here we present SMITH, an open-source system for sequencing machine information tracking and handling that was developed to meet the demands of the joined sequencing facility of the Italian Institute of Technology and the European Institute of Oncology. SMITH is available at [16] (http://cru.genomics.iit.it/smith/).

## Methods

SMITH has been developed using Java Enterprise technology on the NetBeans 7.3 Integrated Development Environment [17] and runs on a Java EE application server (for instance Glassfish version 3.1 or higher). Apache Maven [18] is used as a software management tool.

The SMITH architecture is divided into a web-tier, a middle-tier, an information system tier, and adheres to the Model-View-Controller (MVC) paradigm. The web interface is provided using Java Server Faces (JSF) [19] and PrimeFaces [20] technology. The FacesServlet, that is part of the JSF framework, plays the role of the Controller and coordinates the information exchange between the user and the Model via a number of views that are provided as xhtml facelets. The Model is composed of JSF Managed Beans that communicate with the information system tier that relies on the Hibernate object/relational mapping [21] to communicate with a MySQL database. The messages displayed are organized in a resource bundle for allowing easy internationalization.

SMITH generates dynamically the scripts that perform the initial data processing (up to FASTQ files). Further analysis of FASTQ files is performed by the workflows of Galaxy. Finally, SMITH communicates with the NGS data file system via a mount point in the application server and can launch commands on a high performance computing cluster (HPC) to run the various workflows. This design has proven stable and scalable to multiple users.

## Results

### Infrastructure around SMITH at the Center for Genomic Science

**Figure 1:**
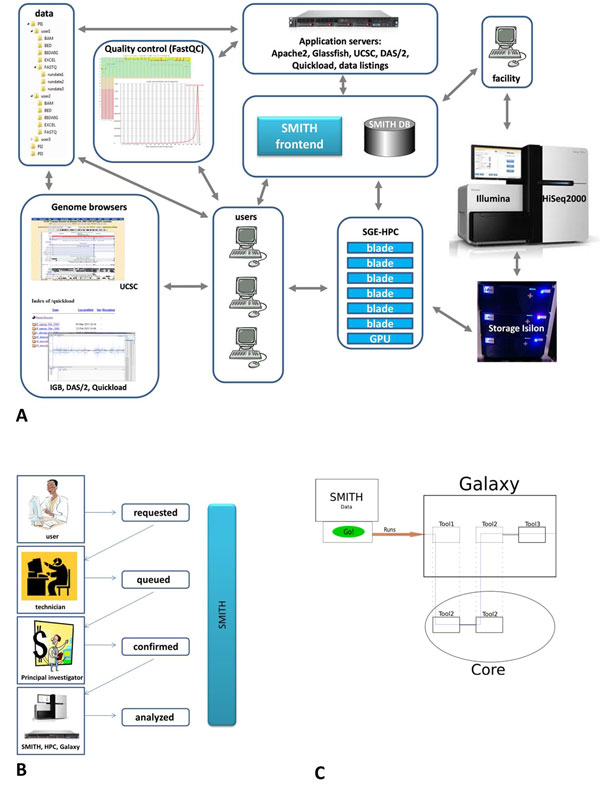
**Infrastructure, main tasks, and architecture**. A) Infrastructure: Sequencing is performed on an Illumina HiSeq2000 instrument. Data are stored on an Isilon mass storage device. Data are elaborated on a Sun Grid Engine High Performance Computing cluster (SGE-HPC). Application servers run web applications for Genome browsing, data listings, the SMITH LIMS, and host the MySQL information tier. The user data directories are organized by group leader name, user login name, file-type, and run date. B) Sample tracking in SMITH. A sample passes through four states ("requested", "queued", "confirmed", "analysed"). Submitted samples have status "requested". When a sample is added to the virtual flow cell, its status changes to "queued". Upon the group leader confirmation the status changes to "confirmed". The sample is then run and analysed by the workflow engine and assumes the final status "analysed". HPC refers to a high performance computing cluster. C) Architecture of the workflow unit. Generated commands invoke Galaxy workflows that subsequently call the un-pluggable core. A part of the instruments can be on the Galaxy side (proprietary tools and scripts) and the other part (open-source tools) is moved to the core.

Before presenting SMITH in detail, we briefly describe the infrastructure that SMITH operates in (Figure 1A). The IIT Genomic unit sequences about 2000 samples per year on an Illumina HiSeq2000 instrument submitted by 150 users belonging to 20 different research groups. The raw data are first written to a RAID5 configured set of hard disks on a local workstation running the HiSeq2000 controller software and contemporaneously transferred to a remote Isilon mass storage device as the sequencing cycles proceed. This buffering procedure precludes data loss due to temporary network failures in the connection between the workstation and the Isilon device and guarantees having the raw data entirely copied to their final destination in near real time. Upon finishing the chemistry cycles, the data are de-multiplexed and converted to FASTQ format using CASAVA software. FastQC software [22] is used for quality control. The FASTQ files are then distributed to individual users for further analyses. The user directories are organized by group leader's name, user name, file type, and run date to facilitate access to the data. FASTQ files are stored in the FASTQ directory, BAM files in the BAM directory, etc. (Figure 1A). Each file has two links (generated using the UNIX ln command). The two links permit access to the same data file from two different locations. The first link is located in the user directory as described above. The second link is found in the CASAVA output folder and facilitates access to the data using the flow cell barcode, the run date, or the run number by facility staff. The FASTQ data are being analysed further either by individual group bioinformaticians or subjected to SMITH workflows, as requested by the user. All computation intensive tasks are performed on a Sun Grid Engine High Performance Computing (SGE-HPC) cluster that has mounted the Isilon data directories. Analysed data can then be viewed using a local UCSC Genome browser mirror or other genome browsers such as the Integrative Genomics Viewer (IGV) [23] or the Integrated Genome Browser (IGB) [24]. IGB users can take advantage of a DAS/2 server [25] and a Quickload directory installation [26] for easy access to a large variety of genomes and annotations.

### SMITH main tasks

SMITH orchestrates the data flow from the user request up to the FASTQ/BAM/BED data. The main tasks and features are:

• Sample submission

• Sample annotation

• Sample analysis

• Sample tracking

• Project awareness

• Run folder monitoring

• Quality control

• Reagent storage

• Role-based access

• Virtual flow cells

• Index compatibility testing

• Email alerts

The users interact with SMITH for placing a sequencing request, for choosing sequencing and analysis options, for adding sample descriptions as key value-pairs for later meta-data analysis, and for tracking their samples using the SMITH web interface. Users can also arrange samples into projects and choose collaborators for role-based access to the data. The facility staff interact with SMITH for assembling flow cells, for inserting reagent barcodes, and for keeping track of reagent stocks.

SMITH offers assistance in assembling flow cell lanes by choosing samples with compatible barcode index sequences to avoid barcode collisions at the de-multiplexing stage allowing one or two mismatches. From the user point of view, the most important features are sample submission and sample tracking. Figure 1B shows the states a sample assumes as it is passing through the sequencing pipeline. Submitted samples have sample status "requested". Before starting a new run, a virtual flow cell is generated and distributed to the group leaders involved for final approval. Samples added to the virtual flow cell change their status from "requested" to "queued". The confirmation by the group leader changes the sample status to "confirmed". Once a flow cell is run, a sample sheet needed for de-multiplexing is generated automatically. When a finished run is detected, the processing engine generates and executes all the commands that are necessary to generate FASTQ data, copy the data to user directories, run FastQC, initiate Galaxy workflows for further processing and send an email alert to the user that their data are now available. At this point, the sample status changes to "analysed".

### Role-based access

The role-based access was implemented in order to minimize data management errors. Moreover, the role-based framework allows controlling data visibility. Users can access only data of the projects they are involved in.

SMITH supports the following roles:

• A "user" can add samples, track their state and browse the data. Users have access only to data that belong to their group leader.

• A "technician" can modify information related to sequencing runs (flow cell IDs, lanes, etc.). Technicians have access to all submitted samples.

• A "group leader" has more extended access rights, than a "user". He can access all the data of his group. A group leader confirms assembled flow cells.

• An "admin" has ultimate access. Admins have access to all the data and can add new users.

### Workflow subsystem

After the sequencing run is finished the workflow subsystem performs the data processing steps. The processing is run automatically when the raw data is ready. Alternatively, it can be initiated manually by a user. The workflow subsystem includes two levels: the scripts for initial processing from BCL (base call files) to FASTQ files and the backend processing unit that is described below. The scripts perform de-multiplexing and FASTQ conversion with CASAVA and generate quality control reports with FastQC. The architecture of the main processing unit is shown in the (Figure 1C). This architecture is created to meet two requirements: on the one hand - an extendable workflow system that is able to handle any kind of NGS data processing and on the other hand - an un-pluggable core of the computational tools that can be installed in other facilities with minimal effort and can be used for exploratory analysis. Galaxy has a highly integrative infrastructure and a friendly graphical user interface. Therefore, Galaxy is used as a primary workflow management system. Galaxy allows extending the pipelines by custom tools and scripts but requires extensive configuration. To circumvent this problem, a subset of the tools of the pipelines was integrated into the UGENE Workflow Designer [27], which is subsequently called by Galaxy. Because of the simple structure of the UGENE Workflow Designer, it can be easily unplugged from the system and installed to any other server or personal computer. Moreover, the way that Galaxy processes multiple inputs seems limited to the task of batch processing of NGS data. For instance, there is no direct way to support merging of multiple BAM files through the Galaxy API. That is why these functions are performed by the UGENE Workflow Designer. The current version of the workflow subsystem includes the pipeline that performs the alignment of FASTQ data with BWA [28] and converts the resulting data to BAM and BigWig formats.

From the technical point of view, the workflow subsystem involves the following steps:

• For each NGS run, SMITH generates scripts that include all input files and all commands to be executed during the analysis.

• The commands invoke the Galaxy runner. This is a Python script that uses the Galaxy API with the help of the BioBlend library[16]. The scripts provide the input, wait until Galaxy analysis has finished, and download the results.

• Galaxy partially invokes the UGENE Workflow Designer, as show in Figure 1C.

Since the workflows used in data processing are in constant change, the flexible workflow subsystem can be quickly adapted to the changes. Using the Galaxy-UGENE combination, the workflow subsystem can be extended by custom pipelines from both management systems.

In addition to data processing, the workflow subsystem manages the data structures: It puts the processed data in proper folders and writes the metadata references back to SMITH.

### Data model

The data model of SMITH was designed for achieving the following goals:

• Progressively store all the information produced in the NGS process, from the sample request to the analysis parameters

• Manage the access to the information for all the users, depending on a role and a group

• Adapt to the existing pipelines and data flows in the research centre

• Provide the possibility to add relevant information at any time

**Figure 2:**
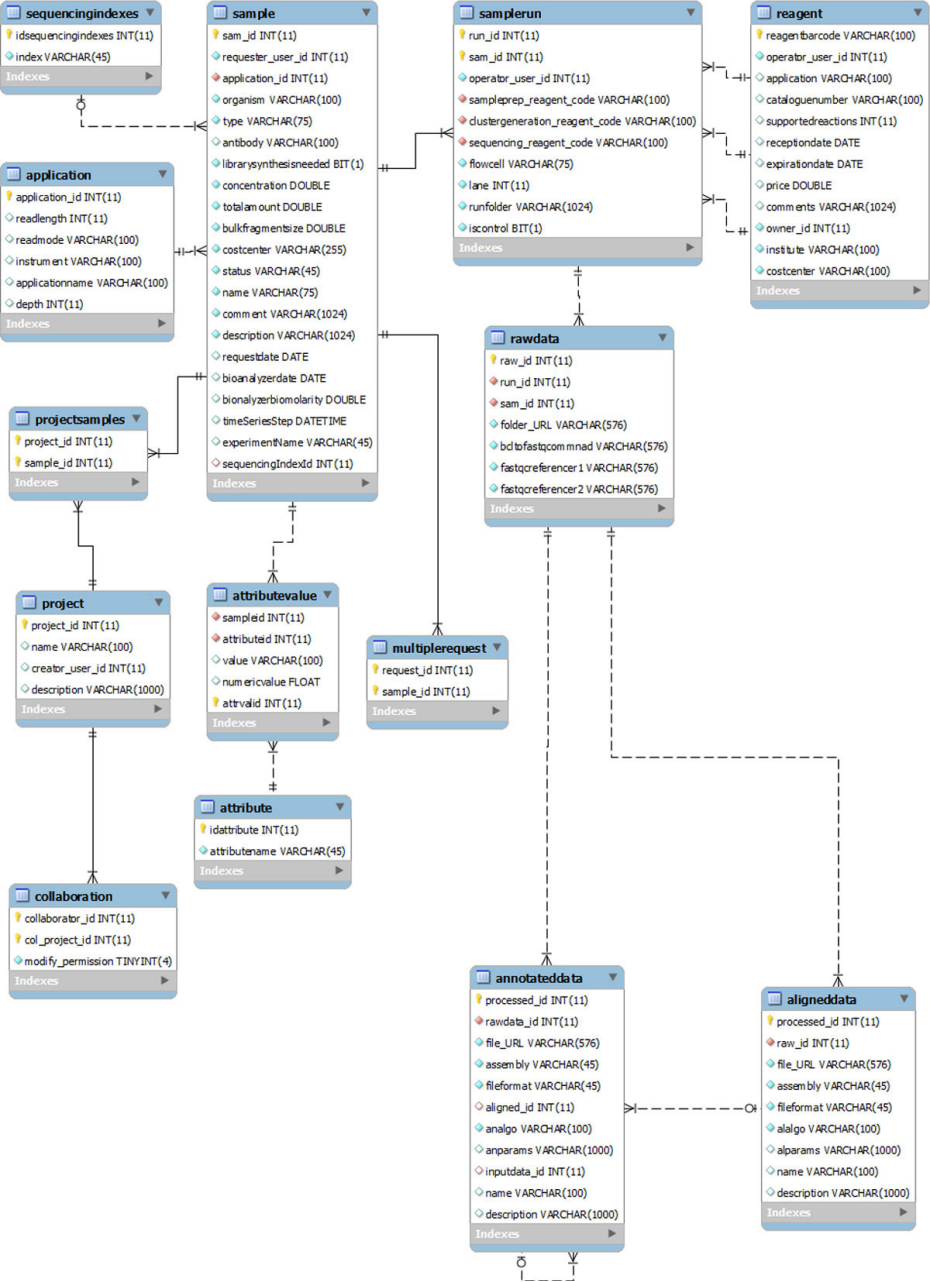
**Data model**. The data model of the SMITH database is shown. The user table is omitted to avoid crossing of table connections. The image was generated using MySQL Workbench software.

In Figure 2 we show the IEEE (Institute of Electrical and Electronics Engineers Standards Association) model of the database. The structure of the database is described in Table 1. To summarize, each step of a sequencing experiment is mapped to the database, from the request to the production of specific files from diverse algorithms. Partially, table records are generated manually by users, especially during the early steps. At later stages, table records are generated automatically by the analysis work flows. The attribute-value structure permits enriching each sample, and thus all the connected entities, with a detailed description that can easily be translated to xml or other formats that might be useful for other analysis purposes (for example clustering algorithms). The possibility to regroup samples into projects facilitates customizing role-based access to the data.

**Table 1:** Description of database tables.

Table	Description
**User **(not shown in Figure 2)	Includes name and surname, phone number, email, etc. The passwords are not stored in the database but provided either from a Lightweight Directory Access Protocol (LDAP) server or from a file realm.

**Sample**	Represents the biological sample of a sequencing experiment. Most attributes are used to set the sequencing machine. A new sample is created at each new request for a sequencing experiment.

**Application**	Contains the parameters characterizing a sequencing run: Read length, read mode, and sequencing depth. These parameters have been combined into a set of predefined recipes. This approach makes it easier for the user to choose appropriate parameters and reduces the number of possible applications, which in turn facilitates sequencing diverse sample in the same sequencing run.

**SequencingIndexes**	Contains all the possible sequencing barcode indices used in the laboratory. When the users prepare their own sequencing library, they must provide information about the sequencing barcode indices.

**MultipleRequest**	Using the web interface, it is possible to request more than one sample at the same time. Such samples are linked by the MultipleRequest table.

**Project**	Groups the samples into projects. A project is associated to a list of users (collaborators). The project creator can set special permissions for collaborators to view or modify the information regarding specific samples.

**AttributeValue**	Connects each sample to custom attributes and values. This approach permits enriching each sample with specific meta-data that can be used for searching for specific samples and for statistical analyses. Note that all the tables connected to sample and representing the results of the sequencing and the following analyses will be connected to the meta-data.

**SampleRun**	Represents the run of the sequencing machine for a specific sample, connected to the sequencing reagents used. Many samples can run together and be connected by the same run_id.

**RawData**	Keep track of FASTQ files produced. It stores the paths to files, samples and runs that originated the data.

**AlignedData**	Stores the algorithm and the reference genome used as well as the path to the resulting aligned data (in BAM format)

**AnnotatedData**	Analysis steps following the alignment are saved in this table. Many algorithms use as input the output of a precedent step. Thus, the table contains a one-to-many reference to itself.

### Advantages of using SMITH

The first version of SMITH had been deployed in September 2011. Since then, more than 4000 samples sequenced on 170 flow cells have been processed. The use of SMITH led to a drastic reduction of the time it takes to carry out primary and secondary data analyses. Primary data analysis refers to de-multiplexing and conversion of BCL to FASTQ format. SMITH automatically generates and launches the scripts needed for primary data analysis. Depending on read length and read mode (single read or paired end read), primary data analysis is generally finished within a few hours after the last chemistry cycle. Secondary data analysis is considerably more complex and our pipelines are in constant evolution. However, the integration with the Galaxy system makes the data available to a wide range of custom scripts.

## Discussion and conclusions

In this study, we described SMITH (Sequencing Machine Information Tracking and Handling), a system for data management in an Illumina sequencing facility that helps to decrease the data-delivery time. SMITH is designed to orchestrate sequencing and data analyses performed by the Genomic Unit of the Italian Institute of Technology (IIT). Utilizing a MySQL database, JAVA EE technology and a Galaxy workflow subsystem, SMITH helps to setup a sequencing experiment, track meta-data artefacts, process raw data and manage data folders. SMITH is a light-weighed, yet effective web-based solution for the management of Illumina sequencing data, which covers the entire workflow from the sequencing request to data presentation in a genome browser compatible data format. Future developments of SMITH will be directed towards taking advantage of the attribute-value pair based description of samples for statistical analyses of in-house generated data as well as for meta-analyses using publicly available data.

## Availability and requirements

The demo version of SMITH is available at [16]. The maintained source code is freely available at [29] under the terms of the MIT licence.

SMITH can be used for the management of NGS experiments derived from the Illumina platform. A system administrator will have to install and maintain a MySQL server and a JavaEE application server. Management of data produced by other sequencing platforms may be possible, although we do not have first-hand experience. Adaptation of SMITH for use with alternative sequencing platforms likely requires a limited set of changes in the script generator, which is tailor-made for Illumina sequencers in the current implementation.

The workflow subsystem can be used independently of SMITH, as a part of Galaxy or UGENE packages. It requires preinstalled workflow management systems and workflow schemes, which are employed by SMITH.

In order to use SMITH for NGS data-connected operations (automatic monitoring of run folders, running workflows, data updating, etc.), the same NGS data and workflow infrastructure (Figure 1A, Figure 1C) must be provided by a host. The customization of SMITH should be discussed with authors in that case. The path to the relevant data folders can be specified as context parameters in the web.xml configuration file. For branding purposes, an institute logo can be displayed. Furthermore, customization of the rendering styles of the template.xhtml will change the appearance of the entire application, if desired.

## Competing interests

The authors declare that they have no competing interests.

## Authors' contributions

FV contributed to the design and testing of the SMITH core system, including interfaces and database management. YV developed the workflow subsystem. AC contributed to the design and testing of the SMITH core system. HM oversaw the design of this study, designed and implemented the working prototype of SMITH and drafted the manuscript. All authors read and approved the final manuscript.

## Declarations

Publication costs for this article were funded by the corresponding author's institution.

This article has been published as part of *BMC Bioinformatics *Volume 15 Supplement 14, 2014: Semantic, social, mobile applications for bioinformatics and biomedical laboratories: selected works from the 13th International Workshop on Network Tools and Applications in Biology (NETTAB 2013). The full contents of the supplement are available online at http://www.biomedcentral.com/bmcbioinformatics/supplements/15/S14.
